# Institutional Needs

**Published:** 1918-06-29

**Authors:** 


### Institutional Needs.

### THE "GODDARD" PATENT HAMMOCK BED.

(PROPRIETOR, F. GODDARD, 30 COLEMAN STREET, LONDON, E.C. 2.)

THIS is a capital improvement on the many forms of camp bed offered in the market. It is light and compact, two great points to the traveller. The first thought nowadays will be "What is its use in war?"

Its uses are many. It is as light as an ordinary stretcher, and can be used as such. The bed would be a valuable addition to the equipment of any medical unit. As a bed it is most comfortable, and as it is so light and handy, the patient, bed and all, could be carried on it from ward to theatre without the need of transfer to a stretcher. Equally easily a patient can be carried out into the grounds round his hospital, hut, or tent, to enjoy fresh air and summer weather. There is one pattern with specially designed wheels fitted, so that one person can run the bed about. It is doubtful, however, if the extra weight and extra packing, though not very much, will be held to be compensated by the extra convenience.

It is not only during the war, but in that vague period "after the war" that the bed is to be used and valued. When men and women may take their ease again, and pass quiet summer days in gardens, no more convenient hammock can be imagined, and no tree for suspension is required. When once again there is leisure for travel and sport, what better for the *shikari* in the Indian hills or the wandering hunter on the East African plains than such a bed as this, only one-third of a coolie load?

